# Beyond predatory journals: peer review transparency as a preventive strategy for editorial integrity in scholarly communication

**DOI:** 10.3389/frma.2026.1850943

**Published:** 2026-08-21

**Authors:** Flavio Aliaga

**Affiliations:** Dirección de Investigación, Universidad Tecnológica del Perú, Lima, Peru

**Keywords:** editorial integrity, open peer review, paper mills, peer review transparency, predatory journals, research integrity, scholarly publishing

## Abstract

Since the emergence of predatory journals, the integrity of scientific communications has faced serious challenges, particularly when this integrity is assessed solely through external indicators which, while providing an initial safeguard, do not ensure in practice whether there was a rigorous, independent peer review, or whether traceable evidence exists regarding the decision leading to acceptance. This study focuses on complementing existing detection systems with transparency in the peer-review process as a preventive strategy against the current opacity of this process in most scientific journals. Furthermore, this structural vulnerability is not limited to clearly deceptive journals but may also be reflected in legitimate journals due to weaknesses in governance, poor oversight, or manipulation by unethical actors, without necessarily implying an explicit intent to engage in misconduct. This transparency alone does not solve the problem of predatory journals, but by intervening at the most critical and vulnerable point, it could be considered a strategy that—through explicit peer review policies (*ex ante*), process traceability (*in-process*), and auditability (*ex post*)—and progressive or full disclosure of review reports—can prevent anomalies or mitigate the impact of malpractice on editorial integrity. Finally, this study does not seek to replace existing predatory journal identification systems but to complement the verification of editorial processes with strategies that are integral and genuine, ensuring trust and truth in scientific communications.

## Introduction

In recent decades, scientific communication has undergone global expansion and diversification, driven largely by open access, international cooperation, and publishing digitalization (Piwowar et al., 2018; Leung et al., 2023; Maier, 2025). This expansion reflects not only growth in the number of scientific publications but also new opportunities for predatory journals to expand their manipulation mechanisms and fraudulent practices, such as cases of fake peer reviews (Bell et al., 2024) and paper mills (Parker et al., 2024), where editorial integrity is compromised through simulated, nonexistent, or deficient peer review processes (Beall, 2012; Elmore and Weston, 2020). In response, automated detection systems or indices for identifying predatory journals have been developed (Chen et al., 2023; Hinze et al., 2026); however, detection based on warning signals may enable unethical actors—a person, group, or organization acting against ethical principles and scientific integrity—to generate new evasion strategies.

Early warning signs are useful for uncovering editorial problems, but they do not allow these characteristics to be turned into criteria that fully define a journal as predatory, nor do they clarify whether the review process was legitimate (Shamseer et al., 2017; Grudniewicz et al., 2019). Likewise, a journal's mere declaration of peer review is neither a sufficient guarantee nor sufficient evidence of a rigorous, independent, or traceable process that documents the decisions made (Ross-Hellauer, 2017; Tennant et al., 2017; Ross-Hellauer and Horbach, 2024). In other words, this effort does not answer the question of *how we can ensure that peer review is rigorous and independent*. *What verifiable trace did the acceptance decision leave*?

This research does not seek to redefine predatory journals but rather to shift the focus to the opacity of peer review as a form of structural vulnerability in the publishing system. It proposes transparency in the review process as a preventive strategy to strengthen credibility, avoid anomalies, and safeguard the integrity of scientific communication.

## What external indicators can—and cannot—show

Warning signs, “*red flags*”—misleading or unverifiable editorial information, fictitious editorial board, extremely rapid publication times, disproportionately broad scope of the journal, lack of clarity about publication costs, unsolicited mass emails aimed at attracting authors and manuscripts, and website impersonation (hijacked journals)—are external indicators associated with the detection of potentially predatory journals and play an important role in the preliminary assessment (Elmore and Weston, 2020; Abalkina, 2024). In fact, the main utility of these indicators is to provide alerts for a more detailed evaluation of the rigor, traceability, and best practices in the editorial processes claimed by journals (Shamseer et al., 2017; Grudniewicz et al., 2019). Additionally, Shamseer et al. (2017) identified 13 important distinguishing characteristics; however, they were unable to link them to an absolute definition of predatory journals. Therefore, these indicators should be handled with caution as they may lead to false positives; that is, some of these indicators could be observed in legitimate journals that may be affected by authors who falsify data without clearly indicating intent by the editor or journal to engage in malpractice. This shows that the identification and implementation of detection systems (Chen et al., 2023; Hinze et al., 2026) should not be the only response to the problem of predatory journals. Rather, external detection should be complemented by internal verification of the process, strengthening the authenticity of the declared peer-review process and preventing new unethical mechanisms that undermine editorial integrity.

## Threats to editorial integrity across publishing systems

The violation of editorial integrity is intensified in high-risk editorial systems, where the opacity of their processes allows questionable practices to filter through, affecting various actors in the publishing ecosystem (Gasparyan et al., 2015; Moher et al., 2017; Candal-Pedreira et al., 2022). This has also been observed in legitimate journals indexed in recognized databases through discontinuation and systematic retractions due to manuscript fabrication or reviewer manipulation (King, 2024; Kincaid, 2023a,b; Parker et al., 2024). The publication of fabricated articles or those accepted through simulated, manipulated, or superficial peer review processes not only undermines the credibility and reputation of journals but also degrades their scientific evidence and, if applicable, grants undue academic merit (Parker et al., 2024; Candal-Pedreira et al., 2022) and possible financial incentives. Consequently, peer review is an extremely critical process whose opacity limits the verification of how genuine, independent, and rigorous the assessments have been and exposes the system to potential structural breaches (COPE, 2017; Ross-Hellauer, 2017). Various manipulation schemes have exploited this opaque structural process:

1. *Paper mills*: This is a structured and systematized organization of unethical practices focused on the production and commercialization of articles, data, and authorship to insert them through deceptive or manipulative practices, as well as through deficiencies in journals' editorial review and oversight processes. Mass retractions were associated with this phenomenon (Kincaid, 2023a,b; King, 2024), where legitimate journals indexed in reputable databases and with relevant metrics were also involved (Candal-Pedreira et al., 2022; Abalkina and Bishop, 2023; Parker et al., 2024; Mascato-Fontaíña et al., 2025), demonstrating that this serious problem is centered on the peer review process.
2. *Fake peer review*: This occurs when journals are induced to accept fake reviewers or fabricated identities—using email addresses controlled by the authors themselves or other intermediaries—which produces simulated assessments that facilitate favorable decisions without robust scrutiny or real content evaluation (Drazen et al., 2015; Oransky, 2021).
3. *Special issues*: This is an editorial scheme designed to produce legitimate publications relevant to specialized topics; however, it can become a vulnerable gateway when editorial oversight, robust verification of evaluation processes, and integrity controls are insufficient, making it easier for deficient papers or even articles linked to paper mills to slip through (Kincaid, 2023a,b; Dainton and Dixon, 2024; King, 2024). Additionally, the impersonation of guest editors has led to the retraction of entire special issues and even the closure of the journals involved (Oransky, 2021).

## Peer review as a high-risk black box in scholarly communication

Peer review must ensure a rigorous and independent evaluation process, considering that the reviewer's expertise or specialization matches the subject of the submitted manuscript (COPE, 2017; Tennant et al., 2017; Horbach and Halffman, 2018). However, the editorial design of many peer review models is characterized by high opacity, as reports are rarely made public, evaluation criteria are not always explicitly stated, and the process can hardly be verified by the scientific community (Ross-Hellauer, 2017; Bravo et al., 2019). Consequently, when an article is published, the process that led to that decision generally remains a “*black box*,” that is difficult to reconstruct and critically assess. This peer review with limited transparency suggests that strategies aimed at addressing these issues should not be limited to journal classification or detection of individual cases, but rather to incorporating transparency into the process as a structural principle of the editorial system (Ross-Hellauer, 2017; Bravo et al., 2019; COPE et al., 2022; COPE Council, 2025a).

## A preventive strategy: how peer-review transparency can operate

Transparency in peer review does not, by itself, solve the problem of predatory journals but directly intervenes in the process by which it is decided which works are accepted and enter the scientific record; that is, it acts at a critical point where the system is most vulnerable to predatory journals. Therefore, beyond simply questioning which journals are suspect, it is essential to examine the information they provide about how manuscripts are evaluated, who participates in these decisions, and what traces are left by editorial processes (Ross-Hellauer, 2017). Within this framework, open peer review models can be understood as a set of practices aimed at making a journal's evaluative system visible and expanding the available information on their reviews, arguments, and decisions (Ross-Hellauer, 2017; Tennant et al., 2017). Ross-Hellauer (2017) describes open peer review as an “umbrella” term that encompasses several dimensions of openness, including the publication of reports, disclosure of identities, and availability of underlying data. However, not all these dimensions carry the same weight in the face of editorial integrity breaches; in this context, the visibility of review reports and traceability of the editorial history of manuscripts are fundamental (Ross-Hellauer, 2017; Ross-Hellauer and Horbach, 2024). Moreover, publishing these reports would not only allow for observing the nature of objections and the depth of analysis, but also the coherence between recommendations and the final decision, potentially reducing fabricated reviews, purely formal evaluations, or arbitrary decisions (Bravo et al., 2019; Ross-Hellauer, 2017), and could also help limit the silent infiltration of manuscripts from paper mills and the simulation of unethical processes (Candal-Pedreira et al., 2022; Parker et al., 2024; Mascato-Fontaíña et al., 2025). On the other hand, surveys of open peer review showed favorable results regarding the publication of these reports but also revealed little support for disclosing identities (Ross-Hellauer, 2017; Ross-Hellauer and Horbach, 2024), which could cause some reviewers to exercise caution in their language, soften their criticism, or decline invitations in sensitive situations (Bravo et al., 2019). Nonetheless, Nature Communications (2016) created a system called transparent peer review, where the publication could attach both review reports and authors' responses, keeping reviewer identities anonymous unless they chose to sign their reports. Therefore, rather than imposing a single model, it is reasonable to move toward gradual schemes—for example, open access reports with optional reviewer signatures—accompanied by explicit policies on the review model and processes for correction and retraction (COPE et al., 2022; COPE Council, 2025a).

**Figure 1:**
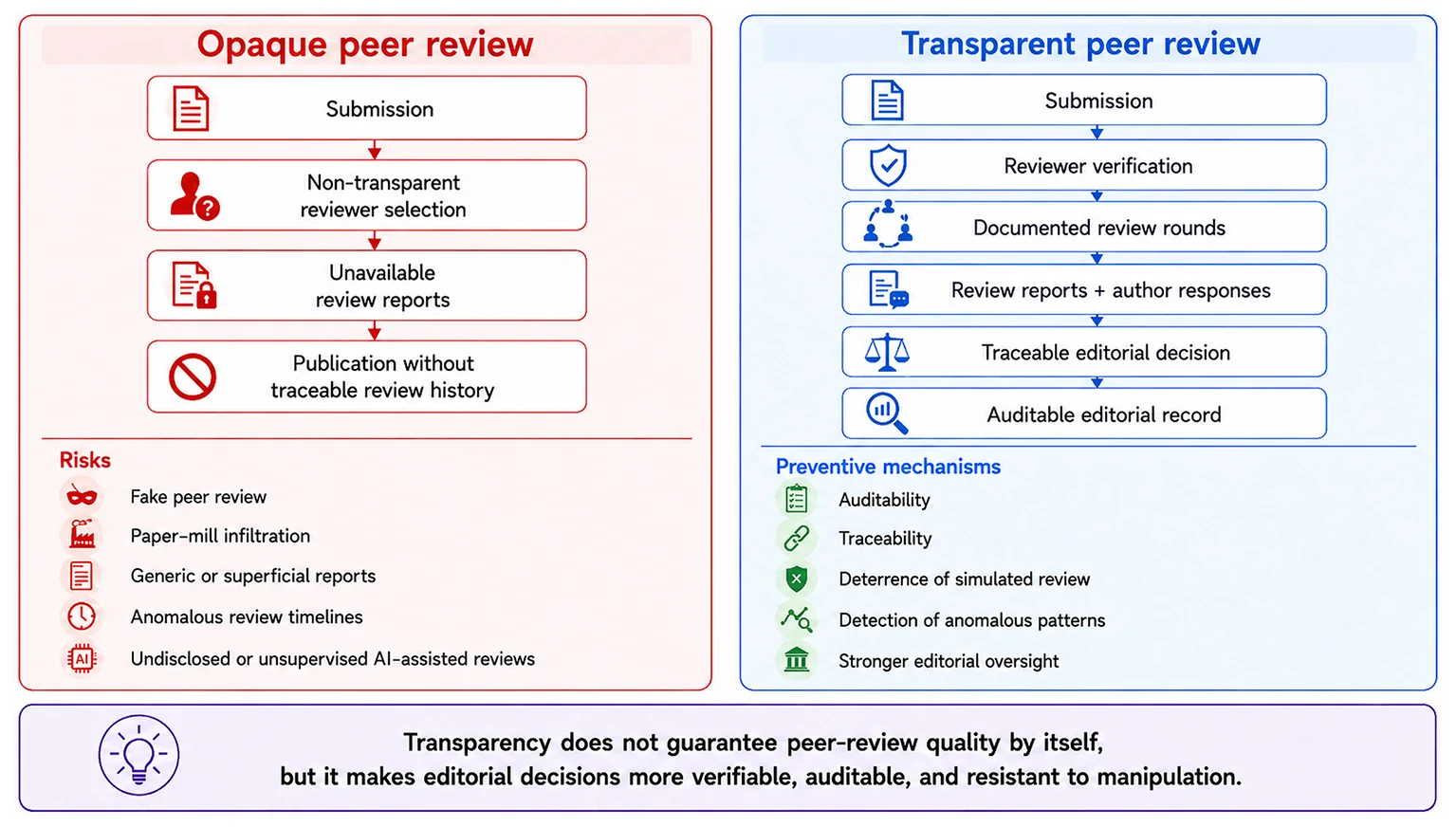
From opaque peer review to editorial transparency. On the one hand, the risk of opaque peer review exposes the documented vulnerability in an environment accessible to the infiltration of paper mills, fake reviewers, insubstantial reviews with anomalous timelines, and misleading statements about the use of artificial intelligence **(Left)**. On the other hand, one response to this problem is peer review transparency, creating auditable and traceable mechanisms, safeguards against simulated reviews, early alerts through the detection of anomalous patterns, and increased integration of editorial oversight in all processes **(Right)**.

Preventive strategies can mitigate the risks of unethical practices against editorial integrity (Figure 1); however, these preventive actions would not be fully effective if they depended solely on publishing review reports after acceptance. In this sense, considering existing recommendations on describing transparency, ethical and documentary management, and openness of peer review (COPE et al., 2022; COPE Council, 2023, 2025b; ICMJE, 2026), a preventive framework is proposed and organized into three complementary stages:

1. *Ex ante*: The strength of any preventive strategy lies in preparing a foundation of actions before the event occurs; that is, as a starting point, journals should clearly report which peer review model they will use, how confidential information and conflicts of interest will be managed, the distribution of editorial responsibilities, rules on the use of artificial intelligence, proper verification of reviewers and guest editors in special issues under the supervision of the editor-in-chief, and which documents might be made public. Although these measures do not completely eliminate the risk of editorial process manipulation, they can make it more difficult to impersonate reviewers and guest editors (COPE Council, 2023, 2025b).
2. *In-process*: During this stage, transparency should be framed in the generation and preservation of editorial files for each manuscript. This record should include the dates for each stage of the process, all versions of the manuscript, review reports, associated correspondence, and a record of the use of artificial intelligence tools. The traceability generated at this stage becomes an opportunity to detect signals that require additional verification, such as extremely rapid evaluation times, recurring reviewers, unverifiable addresses, and poor-quality review reports, although not as conclusive proof of manipulation (COPE Council, 2018; ICMJE, 2026).
3. *Ex post*: After the decision to accept and publish the article, the journal could adopt gradual schemes or provide full disclosure of review reports, authors' responses to comments raised, and editorial decisions as additional documents available to the scientific community. Furthermore, beyond demonstrating the basis for editorial decisions and any detected anomalies, such auditability could help discourage fabricated reviews and other bad practices that are contrary to editorial integrity.

## Discussion

The umbrella-type open review is characterized by grouping together forms of openness, such as the publication of reports and disclosure of reviewers' identities (Ross-Hellauer, 2017); however, the publication of these reports elicited a more positive attitude than the identification of reviewers (Ross-Hellauer and Horbach, 2024). This article concurs with advancing open science but considers the possibility of using transparency as a mechanism to verify the editorial process rather than focusing on it as a criterion for determining whether a journal is predatory. In this regard, the joint integration of journals with explicit and traceable policies regarding the review model employed, publishers with improved control mechanisms to ensure integrity at each stage, and academic evaluation systems with verification criteria that complement—not replace—indicators of predatory risk (Bell et al., 2024; Parker et al., 2024; Ross-Hellauer and Horbach, 2024), would enable a more transparent publishing ecosystem, in contrast to the mere use of predatory journal detection tools (Shamseer et al., 2017; Chen et al., 2023; Hinze et al., 2026), which are insufficient when faced with an opaque editorial process that makes it difficult to discern how evaluations were actually conducted and what decisions led to the publication of articles. The opacity of this process can be observed in clearly deceptive journals and legitimate journals with weaknesses in governance and/or manipulation of their review processes (Bell et al., 2024; Parker et al., 2024). Transparency in peer review can be seen as a cornerstone for verifying the proper process, an obstacle to deceptive simulations, the real legitimacy of publications, and a preventive strategy to complementarily strengthen editorial integrity (Ross-Hellauer, 2017; Bravo et al., 2019; COPE et al., 2022), or as a logic similar to a pass-list, but not as a closed approval list; rather, as a verification mechanism based on transparency, traceability, and auditability to deter simulated, deceptive, or deficient editorial practices.

## Conclusion

The problem of predatory journals cannot be addressed solely through the detection of red flags, since these external indicators help identify warning situations that require additional verification in cases of suspected editorial malpractice, but do not reveal what decisions led to the selection of reviewers, the rigor of the evaluations carried out, or the final criteria in the acceptance rulings for the publication of articles. In light of this, this study proposes complementing existing identification systems with transparency in peer review, which could be considered a preventive strategy for verification, traceability, and auditability—progressive or full disclosure of review reports and clear records of the editorial process—to create a deterrent effect against unethical actors in the system and help mitigate the risk of editorial harm from malpractice to the integrity of scientific communication. Finally, this perspective places peer review transparency on the agenda to contribute to editorial oversight and control mechanisms and to further studies on the subject, enabling the consolidation and safeguarding of trust, truth, and integrity in scientific publications.

## Data Availability

The original contributions presented in the study are included in the article/supplementary material, further inquiries can be directed to the corresponding author.
